# Nothing but Luck

**Published:** 1897-12

**Authors:** 


### Nothing but Luck,

Hard Luck is almost a synonym for laziness.

Good Luck is the twin brother of hard work.

Luck walks, while work rides in a carriage.

Luck pictures a dollar, while work earns it.

Luck dreams of a home, but work builds one.

To trust to Luck is like fishing with a hookless line.

Luck is a disease for which hard work is the only remedy.

Luck longs for a dinner, while labor goes out and earns one.

Luck goes barefooted, while work never lacks for a pair of shoes.

Luck is a weather vane with the distinguishing points broken off.

The man who relies on Luck is lucky if he keeps out of the poorhouse.

